# Assessment of Medication Adherence in Saudi Patients With Type II Diabetes Mellitus in Khobar City, Saudi Arabia

**DOI:** 10.3389/fphar.2019.01306

**Published:** 2019-11-08

**Authors:** Khaled AlQarni, Elham A. AlQarni, Atta Abbas Naqvi, Dhfer Mahdi AlShayban, Syed Azizullah Ghori, Abdul Haseeb, Mohamed Raafat, Shazia Jamshed

**Affiliations:** ^1^College of Clinical Pharmacy, Imam Abdulrahman Bin Faisal University, Dammam, Saudi Arabia; ^2^Obstetrics and Gynecology, King Fahd Military Medical Complex, Dammam, Saudi Arabia; ^3^Department of Pharmacy Practice, College of Clinical Pharmacy, Imam Abdulrahman Bin Faisal University, Dammam, Saudi Arabia; ^4^Department of Clinical Pharmacy, College of Pharmacy, Umm Al Qura University, Makkah, Saudi Arabia; ^5^Department of Pharmacology & Toxicology, College of Pharmacy, Umm Al Qura University, Makkah, Saudi Arabia; ^6^Department of Pharmacy Practice, Kulliyah of Pharmacy, International Islamic University Malaysia, Kuantan, Malaysia; ^7^Qualitative Research-Methodological Application in Health Sciences Research Group, Kulliyyah of Pharmacy, International Islamic University Malaysia, Kuantan, Malaysia

**Keywords:** medication adherence, diabetes mellitus, type II diabetes mellitus, Khobar, Saudi Arabia

## Abstract

**Objective:** Medication adherence is defined as taking medications as advised and prescribed by health care professionals for stated duration. Diabetes mellitus (DM) is one of the most common chronic illnesses in Saudi Arabia. This study aimed to document medication adherence in Saudi patients with type 2 diabetes.

**Methods:** A quantitative cross-sectional study was conducted in Saudi out-patients with type 2 DM in the city of Khobar, Saudi Arabia. The study used the General Medication Adherence Scale (GMAS) to document medication adherence in this population. Data was analyzed through SPSS version 23. Study was ethically approved.

**Results:** Data was collected from 212 patients. Few patients (35.8%) had high adherence to anti diabetic medications. The correlation between Hb_A1c_ level and adherence score was negative and significantly strong (ρ = -0.413, p < 0.0001). Most patients (N = 126, 59.4%) modified their medication therapy during month of Ramadan and on Eid occassion. Education level was not a determinant of adherence in this population.

**Conclusion:** This study highlighted that medication adherence is influenced by religious and social factors. Patient counseling is required to improve patient beliefs and increase awareness of adhering to prescribed anti diabetic pharmacotherapy. A pharmacist can play constructive role of a disease educator and patient counselor.

## Introduction

Medication adherence can be defined as taking medications as advised and prescribed by healthcare professionals for the stated duration (Osterberg and Blaschke, 2005). It is one of the challenging global issues; non-adherence to medication results in active disease progression and increased treatment costs (Abbas et al., 2015; Naqvi et al., 2019a). According to the literature, almost 50% of the patients suffering from chronic illnesses do not adhere to their medication regimen and half of them indulge in non-adherence after a year of treatment (Haynes et al., 2008). This non-adherence further results in disease related complications and comorbidities that may increase the frequency of hospital admissions, emergency visits and direct treatment costs. These direct costs, depending upon the healthcare sector of a country, may either be borne by the health sector or in some cases the patient (Hussain et al., 2014; Zullig et al., 2015).

The issue of adherence to medication is important for chronic illnesses such as type II diabetes mellitus (DM) as it is characterized by fluctuating blood glucose levels. This fluctuation can either lead to hyperglycemia, i.e., a condition in which there is an elevation in serum level of blood glucose and vice versa, i.e., hypoglycemia (Abbas et al., 2015). Both are detrimental for a patient’s health and well-being. Studies report that uncontrolled DM may lead to macro and micro vascular complications and comorbidities such as hypertension, kidney disorders, retinopathy and in rare cases, Parkinsonism (Abbas, 2014a; Brundisini et al., 2015). Hence, proper management of the disease is helpful in attainment of a healthy lifestyle (Brundisini et al., 2015).

Diabetes mellitus is one of the most common chronic diseases in Saudi Arabia. According to official figures, the incidence rate is positively correlated with age and it is reported at 7.8% and 50.4% among adults and geriatrics respectively (Saudi Health Information 2013; Abbas et al., 2015). The overall disease prevalence in the country is 13.4% (14.8% in males and 11.7% in females) (Saudi Health Information, 2013). World Health Organization (WHO) reports that it is the 5^th^ leading cause of death in the country and with a crude death rate of 4.6% that has increased during last decade (World Health Organization (WHO), 2012). These figures highlight a growing need for managing the illness with proper treatment and adherence strategies (Al-Nozha et al., 2004; Alqurashi et al., 2011;Khayyat et al., 2017).

A study was conducted in Al Hasa district in Eastern Province of Saudi Arabia that reported a high percentage of non-adherence to anti diabetic medications (65–69%) in patients attending a diabetic clinic (Khan et al., 2012). The study documented the finding based on interviews and did not employ a validated research tool to document the practice. Apart from this study, there is a scarcity of published research literature.

This study was conducted to document medication adherence among Saudi patients in the city of Khobar located in Eastern Province of Saudi Arabia, using the General Medication Adherence Scale (GMAS) (Naqvi et al., 2018a; Naqvi et al., 2019b; Naqvi et al., 2019b). By documenting the adherence pattern of type II diabetic patients in Khobar, it is hoped that this study will form the basis for further investigations exploring patient behavior towards adherence.

## Methods

A quantitative cross-sectional study was conducted in a random sample of Saudi patients with type II diabetes, using a validated survey questionnaire to document medication adherence.

## Venue and Duration of Study

The venue of the study was the city of Khobar which is located in the Eastern Province of Saudi Arabia. The Eastern Province is the largest province by area. The city of Khobar is located in the third largest metropolitan area in Saudi Arabia with an estimated population of over 4 million (General Authority for Statistics. Kingdom of Saudi Arabia, 2016). The study was conducted in the out-patient endocrine and diabetic clinics at King Fahd University Hospital which is the largest public-sector tertiary care health facility in the city. The study began in March 2019 and was completed in April 2019.

## Target Population and Eligibility Criteria

The target population for the study was type II DM out-patients. Out-patients who had established diagnosis of type 2 DM (T2DM) and prescribed with oral medications were included in the study. This was done by verification either by medical record, a valid prescription or lab reports. Patients suffering from T2DM alone or with comorbidities such as hypertension, hyperlipidemia, obesity, etc. were included. Comorbidities were identified by an established diagnosis of an illness apart from diabetes. Only Saudi patients were invited to participate, and expatriates were excluded. Patients admitted in hospitals (in-patients) at the time of survey as well as those suffering with acute illnesses were left out. Patients suffering from other phenotypes such as pre-diabetes, type 1 DM and gestational diabetes were also excluded from the study. In addition, patient who were on injectables and those who did not consent to participate in the study and incomplete questionnaires were also not included.

## Sampling and Sample Size

Patients enrolled at the hospital who had appointments in the out-patient endocrine clinics were selected randomly by help of a computer-generated list from hospital’s database. Selected patients were invited to participate in the study by signing a patient consent form. The presence of diabetes was first confirmed from patients and then re-confirmed by their medical record, prescription containing oral hypoglycemic drugs and/or lab results. Following confirmation of illness presence, the patients were asked to provide a random blood sugar (RBS) test for the record. According to official figures from Saudi Ministry of Health, the prevalence of type II DM is 13.4% (Saudi Health Information 2013). Thus, sample size was calculated using the prevalence-based formula:

$$n=Z^{2}P(1-P)/d^{2}.$$

The symbol (n) is the sample size, (P) is the prevalence, (Z) denotes confidence level and (d) is precision (Naing et al., 2006). Substituting the values in the formula we obtained a sample size of 178.

## Research Instrument

The study used the General Medication Adherence Scale (GMAS) to document medication adherence in this population (Naqvi et al., 2018a; Naqvi & Hassali, 2018b; Naqvi et al, 2019c). The GMAS was recently validated in Saudi patients with chronic illness (Naqvi et al., 2019b). The scale is subcategorized into 3 subscales namely, patient behavior related non-adherence (PBNA), additional disease and pill burden related non-adherence (ADPB), and cost related non-adherence (CRNA). The scale has 11 items of multiple choice type (MCQ), and four possible options for each item. Each item awards a score. Each domain of the scale measures a specific dimension of non-adherence. Moreover, GMAS also measures overall adherence to medications. The grading is done based on the scoring criteria, i.e., high adherence, good adherence, partial adherence, low and poor adherence. The scale can provide overall grading as well as grading for each domain for a patient that helps in understanding individual adherence issues. The tool was subjected to reliability analysis using Cronbach alpha and a value of >0.5 was considered satisfactory (Cronbach, 1951; Nunnaly, 1978; Hatcher, 1994). The GMAS with scoring is available from Naqvi and colleagues on request (Naqvi et al., 2018a; Naqvi et al., 2019c).

## Data Coding and Analysis

The data obtained was entered in IBM SPSS, i.e., Statistical Package for Social Sciences version 23 (SPSS Inc. Chicago, IL, USA) software and analyzed. Demographic data was expressed in frequency counts *(N)*, percentages *(%)*. Statistical tests were selected based on data normality. Shapiro Wilk test for normality was conducted to determine data distribution (Shapiro & Wilk, 1965). For non-parametric data, chi square χ^2^ test, and Spearman’s correlation (ρ) were used to report any association of patients’ variables with medication adherence. Significant associations were reported in p values less than 0.05 and correlation coefficients between (-1.0 and +1.0).

## Ethical Approval and Patient Consent

The participants were sought consent before handing the questionnaire. They were briefed about the study objectives and procedure. The participation was voluntary and only those who consented to participate were handed the questionnaire. The study was approved by the Institutional Review Board of Imam Abdulrahman Bin Faisal University, Dammam. (IRB-UGS‐2019‐05‐001).

## Results

The reliability of GMAS was above 0.5, i.e., Cronbach alpha (α) = 0.816. A total of 212 patients responded to the study. The demographic information of respondents revealed that most of the patients were male (N = 142, 67%) and a third were females (N = 70, 33%). Mean age of patients was 44 years (44.17 ± 15.6 years). Besides, most of them were married (N = 156, 73.6%) and had education up to graduation level, i.e., 16 years of education (N = 98, 46.2%). Most patients had a monthly family income above SAR 10,000 (N = 114, 53.8%) and full insurance (N = 126, 59.4%). Half proportion of patients (N = 114, 53.8%) had no comorbidity. Mean Hb_A1c_ was 8.57 ± 2.3. Mean random blood sugar at the time of data collection was 193.4 ± 78.4 mg/dl. Patients had an average of two anti-diabetic medicines per prescription (**Table 1**).

**Table 1:** Demographic information.

Demographic information	Total (N/%)	Male (N)	Female (N)
**Marital status**			
Married	156/73.6	118	38
Single	44/20.8	22	22
Other (Divorced, widowed)	12/5.7	2	10
**Years of education**			
Up to 6 years (Primary)	20/9.4	12	8
Up to 10 years (Secondary)	74/34.9	10	10
Up to 12 years (Intermediate)	20/9.4	52	22
Up to 16 years (Graduation)	98/46.2	68	30
**Monthly family income**			
Less than SAR 5000, i.e., < USD 1332.88	36/17	24	12
Between SAR 5000 to 7500, i.e., between USD 1332.88 to 1999.31.	24/11.3	10	14
Between SAR 7500 to 10000, i.e., between USD 1999.31 to 2665.75.	38/17.9	24	14
Above SAR 10000, i.e., > USD 2666.75	114/53.8	84	30
**Co-morbidity**			
No comorbidity	114/53.8	64	50
Comorbidity present	98/46.2	78	20
**Insurance**			
Full insurance	126/59.4	80	46
Partial insurance	46/21.7	28	18
No insurance	40/18.9	34	6

### Patient Medication Adherence

The mean score for overall adherence to anti-diabetic medications was 26.34 ± 5.6 out of 33. A third of patients were highly adherent (N = 76, 35.8%). Besides, the mean score for patients behavior related non-adherence (PBNA), was 11.61 ± 3.3 out of 15. Half of patients (N = 108, 50.9%) had high adherence. Additionally, the mean score for comorbidity and pill burden related non-adherence (ADPB), was 9.63 ± 2.4 out of 12. Slightly less than half of patients (N = 100, 47.2%) had high adherence. Moreover, the mean score for cost related non-adherence (CRNA), was 5 ± 1.2. More than half of patients (N = 116, 54.7%) had high adherence (**Table 2**).

**Table 2:** Adherence results.

GMAS Adherence scores	Total (N/%)	Male (N)	Female (N)
**PBNA score**			
High adherence (13–15)	108/50.9	74	34
Good adherence (11–12)	32/15.1	20	12
Partial adherence (8–10)	48/22.6	34	14
Low adherence (5–7)	18/8.5	10	8
Poor adherence (0–4)	6/2.8	4	2
**ADPB score**			
High adherence (11–12)	100/47.2	70	30
Good adherence (9–10)	56/26.4	36	20
Partial adherence (6–8)	42/19.8	26	16
Low adherence (4–5)	8/3.8	6	2
Poor adherence (0–3)	6/2.8	4	2
**CRNA score**			
High adherence (6)	116/54.7	86	30
Good adherence (5)	40/18.9	16	24
Partial adherence (3–4)	46/21.7	30	16
Low adherence (2)	10/4.7	10	0
Poor adherence (0–1)	0/0	0	0
**Overall adherence**			
High adherence (30–33)	76/35.8	54	22
Good adherence (27–29)	48/22.6	28	20
Partial adherence (17–26)	74/34.9	52	22
Low adherence (11–16)	10/4.7	4	6
Poor adherence (0–10)	4/1.9	4	0

GMAS, General Medication Adherence Scale; PBNA, Patient behavior related non-adherence; ADPB, Additional disease and pill burden related non-adherence; CRNA, Cost related non-adherence.

There was no statistical association of gender with adherence scores (p > 0.05) except for cost-related non-adherence (CRNA) score, where p value was less than 0.01, (χ2 = 20.84, p = 0.000). In this domain of adherence most male patients had slightly better adherence score compared to females.

### Relationship Between Hb_A1c_ and Adherence Scores

**Figure 1:**
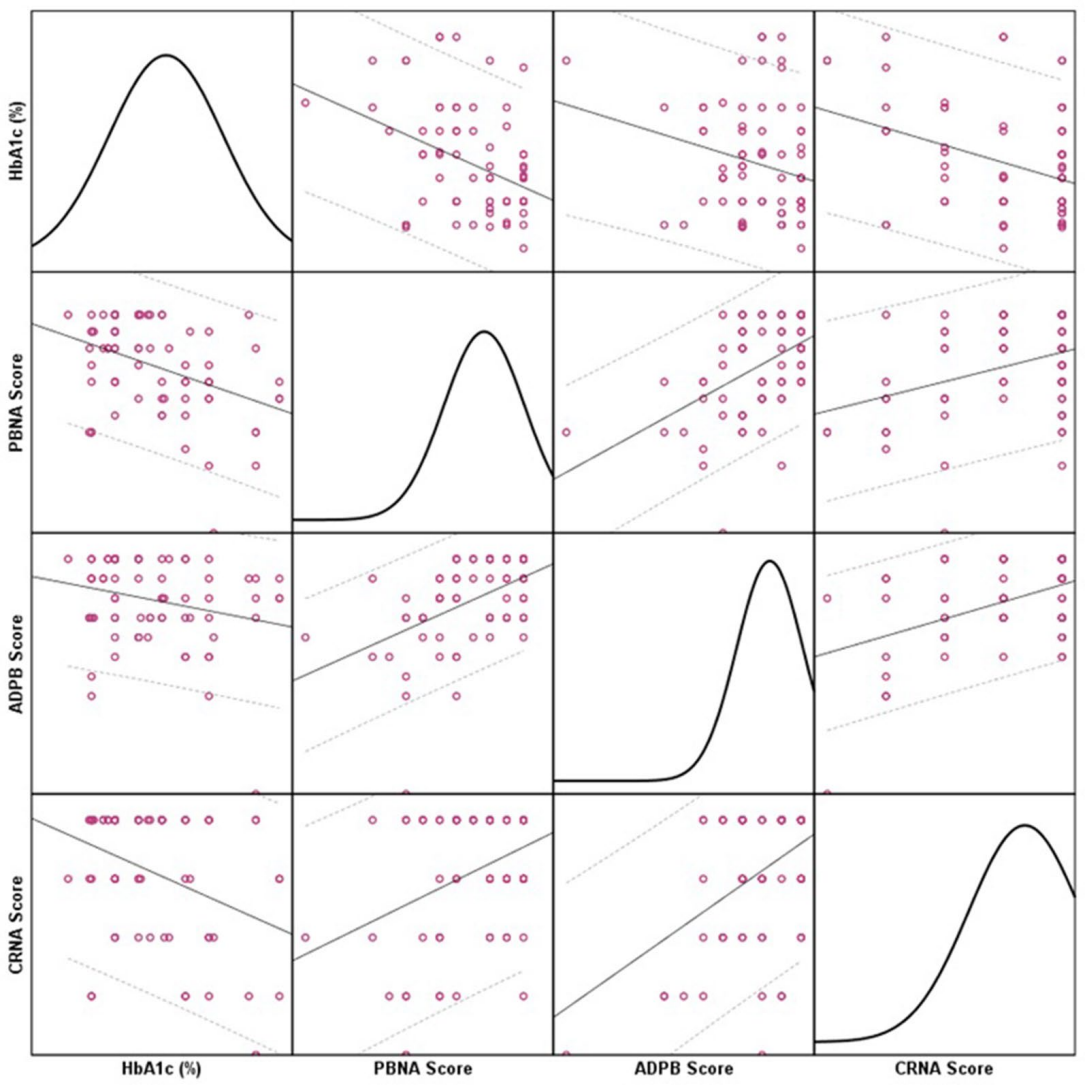
Correlations Between Hb_A1c_ and Individual Adherence Scores.

There was a negative relationship as a significantly moderate-to-strong correlation between Hb_A1c_ and PBNA score (ρ = –0.326, p < 0.01) was reported. Besides, the correlation between glycated hemoglobin and ADPB score was significantly moderate (ρ = –0.231, p < 0.01). Moreover, the correlation between CRNA score and glycated hemoglobin level was significantly moderate-to-strong (ρ = –0.273, p < 0.01). **Figure 1** depicts the relationship between Hb_A1c_ (%) and adherence scores of PBNA, ADPB, and CRNA. The linear line represents relationship while dotted lines represent 95% confidence interval range. The colored circles represent individual patient data. The charts in the diagonal matrices show data distribution.

**Figure 2:**
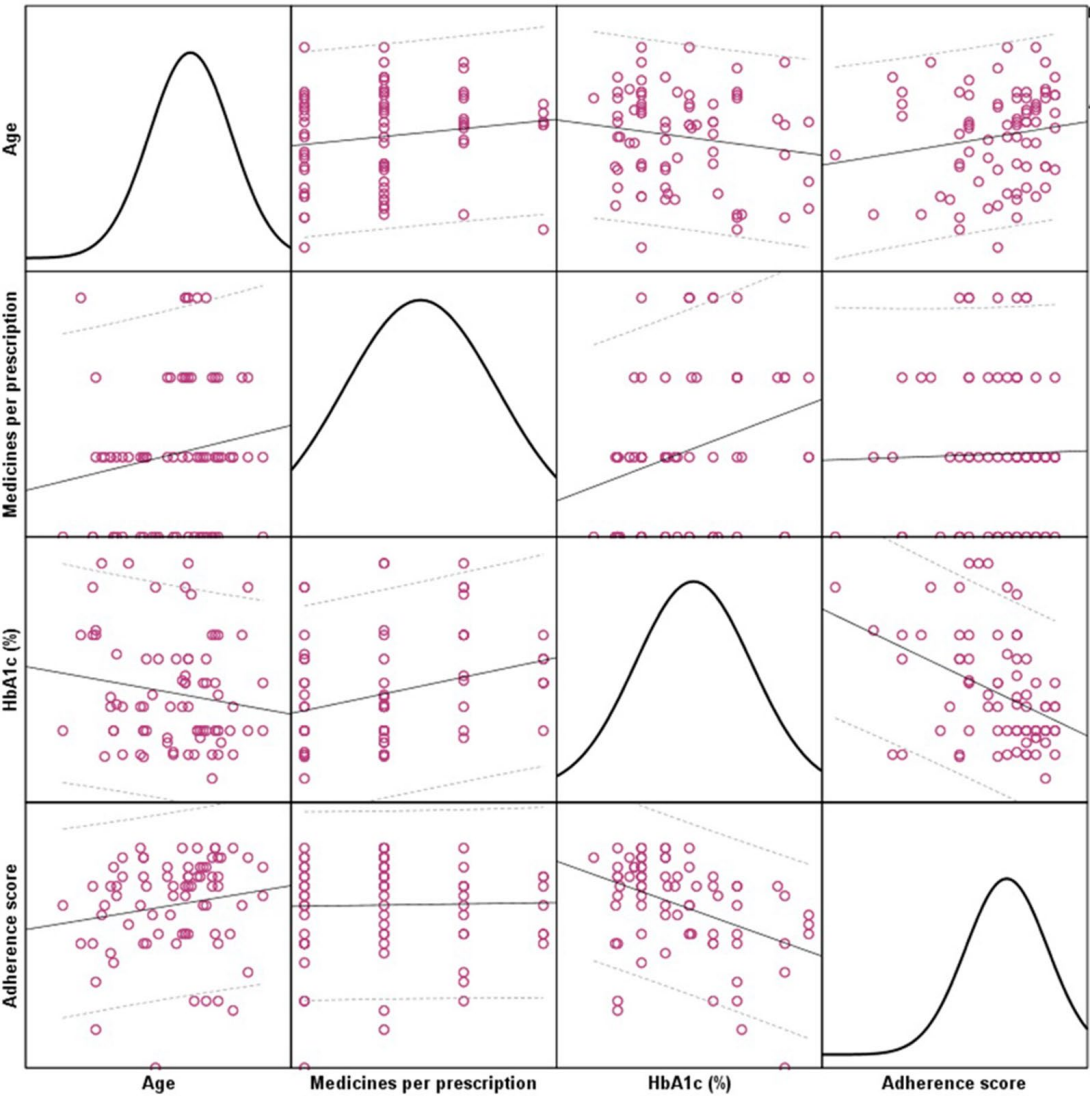
Correlations among age, medicines per prescription, Hb_A1c_ (%) and overall adherence score.

The correlation between Hb_A1c_ level and overall adherence score was significantly strong (ρ = –0.409, p < 0.01) Additionally, patient age was positively correlated with overall adherence score (ρ = 0.193, p < 0.01). The age of patients was also positively correlated with number of medicines per prescription (ρ = 0.183, p < 0.01). **Figure 2** depicts the relationships among age, medicines per prescription, HbA1c (%) and overall adherence score. The linear line represents relationship while dotted lines represent 95% confidence interval range. The colored circles represent individual patient data. The charts in the diagonal matrices show data distribution.

## Discussion

Studies report that the measurement of adherence and patient compliance is quite difficult and most of time is patient dependent (Osterberg and Blaschke, 2005; Brundisini et al., 2015). This study measured adherence to anti diabetic medications in diabetic patients of Saudi Arabia. Most patients were 45 years old. This finding is in concordance with the health statistics of MOH, KSA as it reported high incidence of DM among adult patients in Saudi Arabia (Saudi Health Information, 2013). Only a third of patients had high adherence to their prescribed anti diabetic medications. This figure is lower than those reported by Khan and colleagues in the neighboring district of Al Hasa, i.e., around 70% (Khan et al., 2012). Moreover, the poor compliance to anti diabetic medication therapy was measured by Khattab and colleagues in Abha city of Saudi Arabia and was reported at 1.4%. It was reported by double checking the prescribed and consumed medicines (Khattab et al., 1999;Karim et al., 2014). In comparison with the other countries of the Gulf region, diabetic patients in the UAE had highest self-reported adherence to anti diabetic medications (84%) however, 58.3% of diabetic patients of Palestine were poor to partially adherent to their prescribed regimen (Sweileh et al., 2005). None of these studies employed a validated tool to measure the adherence. In the geographic region around Saudi Arabia, non-adherence to anti diabetic medications among type II diabetic patients of Pakistan was 79.4% (Abbas et al., 2015). All studies reported higher figures compared to our findings.

The medication adherence correlated positively with patient age as our findings suggest that patients tended to be more adherent with increasing age. This could be attributed to the fact that older patients suffer from comorbidities and are supposed to have a greater number of medicines on their prescription (Wu et al., 2012). This was evident in our study as the age of patients was positively correlated with number of medicines per prescription (ρ = 0.383, p < 0.01) and evident in **Figure 2**, i.e., correlation of age with medicines per prescription. This implies that patients in our study were old, had comorbidities and, the number of medicines increased with age. Experience with medication therapy may result in increased awareness regarding adherence. Nevertheless, managing comorbidities with medicines can be a challenging task for geriatrics and studies conducted around the globe reported these as major barriers to medication adherence (Al-Saeedi et al., 2002;Newman-Casey et al., 2015; Jin et al., 2016; Yap et al., 2016). This was also evident in our findings as a quarter of patients had partial-to-poor adherence specifically due to comorbidity and pill burden.

A possible explanation for the low adherence among Saudi patients could be related to their social and environmental barriers. A study conducted by Alsairafi and colleagues in Middle Eastern countries reported that patients of Middle Eastern countries may specifically choose not to adhere to their anti-diabetic regimen as a result of their beliefs particularly ‘fatalism’ (Alsairafi et al., 2016). It is defined as a belief that humans have little control over their lives and all events (including health) are divinely controlled. This belief was found to promote faith with regards to DM management and patients considered adherence a less sensitive issue (Alsairafi et al., 2016). Another finding reported in a study that hampered adherence among patients of Middle Eastern countries including Saudi patients was the uninformed discontinuation or modification of anti-diabetic therapy in the month of Ramadan. Ramadan is the ninth month in the Islamic Hijri Calendar. Muslims around the globe fast in this month from dawn till evening. Taking any food, water or medicine is not allowed during a fast and patients who are on long-term medications have to modify their therapy based on physician’s recommendation (Zainudin et al., 2018). However, it was observed in a study that patient either discontinued or modified their medication dose due to the change in eating pattern without informing their physicians (Alsairafi et al., 2016). Our study also reported that more than a third proportion of patients (42.5%) had forgotten to take medicines in response of religious festivities such as Eid (period of celebration after month of Ramadan).

Evidence indicates that roughly 79% of total Muslim population of the world with T2DM, fast during this month (Salti et al., 2004; Al-Arouj et al., 2010). It is worthwhile mentioning that in Islam, fasting in Ramadan is an obligation for healthy Muslims only. However, studies mention that most patients with diabetes insist on fasting that may be challenging for them. In this regard, the American Diabetes Association (ADA) proposes recommendations for managing diabetes during Ramadan. This includes disease education that is focused exclusively on Ramadan based pharmacotherapy management. Choosing anti diabetic drugs that have lower risk of hypoglycemia and adjusting the doses based on patient’s health profile. There is a need to create an awareness among healthcare professionals as well as patients regarding ADA recommendations for managing diabetes during fasting. This non-compliance could be resolved with educational interventions (Salti et al., 2004; Al-Arouj et al., 2010).

Medication adherence was not associated with educational status of patients as well as monthly family income which implied that educated and uneducated patients had an equal chance of being adherent to pharmacotherapy of DM and vice versa. Studies report a significant relationship between adherence and educational status (Shobhana et al., 1999). However, this is not exhaustive as another study reported that despite being educated and having good knowledge about DM medication therapy, patients appeared to be low adherent to their prescribed regimen (Abbas et al., 2015). This imply that there are some other factors such as social, cultural and personal traits that may influence the medication taking habit of a patient (Shobhana et al., 1999;Wu et al., 2012; Alsairafi et al., 2016). Same explanation could be presented for non-significant association (p > 0.05) of adherence with comorbidities and number of medicines per prescription. A possible explanation to this occurrence, i.e., non-significant association (p > 0.05) of adherence with monthly family income is that most patients had medical insurance. Therefore, it negated the concept of direct medical costs affecting adherence (Naqvi et al., 2018c; Hussain et al., 2014). Hence, there is no out-of-pocket expenditure on purchasing anti diabetic medications. The level of Hb_A1c_ was negatively correlated with adherence score which meant that low adherence was a frequent occurrence in patients with uncontrolled DM.

Gender based cross-tabulation revealed non-significant differences in adherence scores. However, a statistically significant association was observed with cost-related non-adherence (CRNA). Upon further investigation it was observed that most male patients had slightly better adherence score compared to females. Further analyses revealed a significant association between monthly family income and gender (p < 0.03) as well as between marital status and the same (p < 0.03). Most female patients were unmarried and had an average or low income. Whereas, most male patients were married and had higher income. This could be become a social determinant to adherence based on direct cost as female patients who are sole bread earners and who have to support their family financially together with, middle or low income may find it difficult to afford treatment.

The most interesting finding in our study was that, most patients (66%) never tended to discontinue and/or alter their regimen without informing physicians. This behavior could be explained in a number of ways. Since, most patients appeared comorbid and had a high number of prescribed medicines; they may have been educated about the pattern of medicine intake (Royal College of General Practitioners, 2009;Abbas et al., 2015). However, most patients in our study had no out-of-pocket expenditure, comorbidity and high pill burden. According to evidence-based literature on patients’ adherence to medications, it was observed that patients may discontinue their medicines to experience the consequence in order to evaluate its effectiveness (Hussain et al., 2014; Abbas et al., 2015). However, this was not present in our sample as most patients (N = 132, 62.3%) indicated that they never practiced this act. This type of behavior is most seen in comorbid patients having a high medicine count and they may skip medicine/s. Moreover, evidence indicates that patients tend to skip information and may not disclose their non-adherent behavior to physicians (Royal College of General Practitioners, 2009).

According to the American Diabetes Association (ADA) a better Hb_A1c_ is indicative of a better glycemic control over two to four months (Sherwani et al., 2016). Based on this principle we correlated the adherence scores with the glycated hemoglobin values to evaluate their relationship. We observed that the Hb_A1c_ value was negatively correlated with patient’s behavior related non-adherence scores that implied that patients who did not miss their medications based on their behaviors were observed to have better diabetes control. Based on the GMAS scale, PBNA included items related to forgetfulness and deliberately not taking medicines. Besides, the Hb_A1c_ was negatively correlated with comorbidities and pill burden related non-adherence as well. This implied that patients who were punctual in taking medicines in face of comorbidities and pill burden were observed to have better diabetes control. Moreover, the same correlated negatively cost related non-adherence which meant that patients who were adherent to medications based on treatment cost related determinants were observed to have better diabetes control. The three adherence scores were positively correlated with each other and overall adherence score. It implied that all individual scores contributed significantly to the overall adherence of a patient that was a characteristic of GMAS validation (Naqvi et al., 2019b). The highlight of the correlation analyses is that patients with better adherence to anti diabetic medications were likely to have Hb_A1c_ value in acceptable range. This was indicative of a better control of disease (Sherwani et al., 2016).

Pharmacists are the key to address issues of medication adherence especially in non-communicable diseases such as DM (Adnan et al., 2014;Abbas et al., 2015; Naqvi et al., 2019d). In the developed countries, this role is well established. Moreover, apart from Saudi Arabia, community pharmacy practice is established to some extent in other Gulf Arab countries (Alanazi et al., 2016). Saudi Arabia ranks sixth among countries in Middle East and North Africa (MENA) region that have high health care spending. There are over 6000 community pharmacies and more than 22000 pharmacists working in Saudi Arabia (Al-jedai et al., 2016). However, there is an abundance of retail pharmacies that focus on commercial aspects of pharmaceutical services and significantly limits a pharmacist’s clinical role (Alsultan et al., 2013; Haseeb and Elrggal, 2013; Alaqeel and Abanmy, 2015; Mokabel et al., 2017). Besides, 6000 community pharmacies are not adequate considering the health care needs of Saudi population (Haseeb and Elrggal, 2013). Studies conducted to evaluate the working practices at Saudi community pharmacies highlighted deficiencies in medication counseling practices and mentioned the need for improvement (Rasheed et al., 2019). Few studies that have strived to report impact of pharmacists’ led services on patient outcomes in Saudi health sector mentioned that pharmacist counseling services have helped reduce discrepancies in medications during discharge of patients from hospitals, improved clinical parameters such as target blood glucose, and patients were generally satisfied with pharmacists. However, more improvement in pharmaceutical care delivery was required (Al-Tannir et al., 2016; Alfayez et al., 2017; Assiri et al., 2017; Layqah et al., 2018). Hence, betterment in community pharmacy practice could increase patient awareness regarding medication use and translate into better adherence.

Unlike previous literature reported from Saudi Arabia that was limited to reporting of non-adherence rates, our study further highlighted that there was no difference in medication adherence for an educated and uneducated patient (p > 0.05). This highlights the need to counsel patients regarding the importance of adherence. A pharmacist can play an important role of a disease educator and patient counselor in this case (Adnan et al., 2014). Since no difference was seen in adherence levels of educated and uneducated patients, it would be worth mentioning that studies that measure disease knowledge in this population would be helpful if their findings are analyzed in context of adherence. This would highlight if specific diabetes related knowledge has any impact on adherence. Moreover, some novel findings from this study were that although slightly less than half of patients (N = 86, 40.6%) did indicate that they adhere to dosage regimen during religious events and festivities however, most patients (N = 126, 59.4%) did alter their medication schedule due to this phenomenon. Hence, further investigation to explore the impact of fasting in Ramadan and Eid celebration on medication adherence is needed to understand how religious events affects adherence. Further research into exploring patients’ behavior towards medicines intake needs to be investigated qualitatively to have better understanding of particular determinants/factors that may trigger non-adherence in this population.

## Conclusion

Majority of patients had unsatisfactory levels of adherence to anti diabetic medicines. Based on results, it was observed that experience with disease and medication regimen may promote adherence. Patients tended to modify their regimen during festivities such as Ramadan and Eid. Educated and un-educated patients had equal chance of being adherent/non-adherent. There was no relationship between monthly family income and adherence as medicines were available to patients without any out-of-pocket expenditure. This study highlights that medication adherence may be affected by religious and social events in this population. Patient counseling is required to improve patient beliefs and increase awareness of sticking to prescribed therapy.

## Supporting Information

This research paper is based on student research project undertaken as thesis for partial fulfillment of Doctor of Pharmacy (Pharm.D) degree by Khaled AlQarni (ID 2130007246) at College of Clinical Pharmacy, Imam Abdulrahman Bin Faisal University, Dammam 31441, Saudi Arabia. This work is in collaboration with the author of Evidence Based Improvement (EBI) initiative program (Abbas, 2014b; Naqvi, 2016) and supervisors from Saudi and Malaysian universities.

## Data Availability Statement

All datasets generated for this study are included in the article.

## Ethics Statement

The studies involving human participants were reviewed and approved by Institutional Review Board of Imam Abdulrahman Bin Faisal University. (IRB-UGS-2019-05-001). The patients provided their written informed consent to participate in this study.

## Author Contributions

KA, EA, AN, SG, AH, SJ, and DA jointly conceived the idea. AN wrote the introduction with KA, SG, AH, MR, and EA. KA designed the methodology with DA, SJ, MR, AH and AN analyzed data with assistance from SG and EA. KA and EA collected the data and AN entered in the statistical software with DA, SG and AH. AN, MR, SG, AH, and DA wrote the discussion and conclusion with KA. AN, DA, and KA jointly prepared the final draft of the manuscript and carried out editing. The whole work was conducted under the supervision of SJ. All authors read and approved the final manuscript.

## Funding

No funding was obtained for this study.

## Conflict of Interest

The authors declare that the research was conducted in the absence of any commercial or financial relationships that could be construed as a potential conflict of interest.

## Abbreviation

DM, Diabetes Mellitus; WHO, World Health Organization; SPSS, Statistical Package for Social Sciences; SAR, Saudi Arabian Riyal; HTN, Hypertension; MOH, Ministry of Health; KSA, Kingdom of Saudi Arabia; UAE, United Arab Emirates.
